# Marginal structural models using calibrated weights with SuperLearner: application to longitudinal diabetes cohort.

**DOI:** 10.23889/ijpds.v7i3.1783

**Published:** 2022-08-25

**Authors:** Sumeet Kalia, Michelle Greiver, Frank Sullivan, Ervin Sejdic, Michael Escobar, Jessica Gronsbell, Braden O'Neill, Christopher Meaney, Conrad Pow, Olli Saarela, Rahim Moineddin, Tao Chen, Babak Aliarzadeh

**Affiliations:** 1 University of Toronto; 2 University of St Andrews; 3 North York General Hospital

Although machine learning has permeated many disciplines, the convergence of causal methods and machine learning remains sparse in the existing literature. Our aim was to formulate a marginal structural model in which we envisioned hypothetical (i.e. counterfactual) dynamic treatment regimes using a combination of drug therapies to manage diabetes: metformin, sulfonylurea and SGLT-2. We were interested in estimating *diabetes care provision* in next calendar year using a composite measure of chronic disease prevention and screening elements. We demonstrated the application of dynamic treatment regimes using the National Diabetes Action Canada Repository in which we applied a collection of mainstream statistical learning algorithms. We generated an ensemble of statistical learning algorithms using the SuperLearner based on the following base learners: (i) least absolute shrinkage and selection operator, (ii) ridge regression, (iii) elastic net, (iv) random forest, (v) gradient boosting machines, (vi) neural network. Each statistical learning algorithm was fitted using the pseudo-population with respect to the marginalization of the time-dependent confounding process. The covariate balance was assessed using the longitudinal (i.e. cumulative-time product) stabilized weights with calibrated restrictions. Our results indicated that the treatment drop-in cohorts (with respect to metformin, sulfonylurea and SGLT-2) may improve diabetes care provision in relation to treatment naïve cohort. As a clinical utility, we hope that this article will facilitate discussions around the prevention of adverse chronic outcomes associated with diabetes through the improvement of diabetes care provisions in primary care.

